# The ID23-2 structural biology microfocus beamline at the ESRF

**DOI:** 10.1107/S0909049509041168

**Published:** 2009-12-05

**Authors:** David Flot, Trevor Mairs, Thierry Giraud, Matias Guijarro, Marc Lesourd, Vicente Rey, Denis van Brussel, Christian Morawe, Christine Borel, Olivier Hignette, Joel Chavanne, Didier Nurizzo, Sean McSweeney, Edward Mitchell

**Affiliations:** a European Molecular Biology Laboratory, 6 rue Jules Horowitz, BP 181, 38042 Grenoble, France; b European Synchrotron Radiation Facility, 6 rue Jules Horowitz, BP 181, 38043 Grenoble, France

**Keywords:** macromolecular crystallography, automation, microfocus

## Abstract

The first phase of the ESRF beamline ID23 to be constructed was ID23-1, a tunable MAD-capable beamline which opened to users in early 2004. The second phase of the beamline to be constructed is ID23-2, a monochromatic microfocus beamline dedicated to macromolecular crystallography experiments. Beamline ID23-2 makes use of well characterized optical elements: a single-bounce silicon (111) monochromator and two mirrors in Kirkpatrick–Baez geometry to focus the X-ray beam. A major design goal of the ID23-2 beamline is to provide a reliable, easy-to-use and routine microfocus beam. ID23-2 started operation in November 2005, as the first beamline dedicated to microfocus macromolecular crystallography. The beamline has taken the standard automated ESRF macromolecular crystallography environment (both hardware and software), allowing users of ID23-2 to be rapidly familiar with the microfocus environment. This paper describes the beamline design, the special considerations taken into account given the microfocus beam, and summarizes the results of the first years of the beamline operation.

## Abstract 2

Beamline ID23-2, the first dedicated and highly automated high-throughput monochromatic macromolecular crystallography microfocus beamline, is described.

## 1. Introduction

The average size of crystalline samples brought to macromolecular crystallography (MX) synchrotron beamlines has decreased markedly over recent years. Beamlines such as ID23-1 (Nurizzo *et al.*, 2006 ▶) and ID29 at the ESRF regularly receive samples in the 50 µm range which are now considered as standard and for which the beamlines were configured. The driving forces to the use of ever smaller samples come from increasing competition between structural biologists, from increasingly challenging projects which may only lead to tiny crystals and from the realization that in some cases micro-crystals or micrometre-sized portions of larger crystals provide better ordered crystalline lattices and thus improved diffraction quality. Poor quality larger crystals, often being grown from large multimeric protein complexes, may also benefit from small sections of the sample being exposed to a small beam in the search for the best quality data. Beamline ID13 at the ESRF was one of the pioneers in the field of synchrotron microfocus X-ray beams, producing a 30 µm beam in 1994 (Engström *et al.*, 1995 ▶), and numerous results from this facility have demonstrated the benefits of collecting data from micro-crystals with a matched and focused X-ray beam (see references below). Moreover, recently Moukhametzianov *et al.* (2008 ▶), using ID13, published results demonstrating the feasibility of producing good quality diffraction using a 1 µm-diameter X-ray beam and a xylanase crystal. The total crystal volume exposed was around 20 µm^3^. Beamline XO6SA at the Swiss Light Source operates with two set-ups available to users: one delivering a 85 µm × 10 µm highly parallel beam and the second delivering a smaller 25 µm × 5 µm beam to a micro-crystal diffractometer. The changeover between the two set-ups takes about 30 min, allowing users to switch in a single visit. The Diamond I24 dedicated microfocus MAD beamline is also now in operation accepting users since late 2008.

In contrast to these microfocus beams, which provide very high flux densities, Sanishvili *et al.* (2008 ▶) have implemented a mini-beam apparatus capable of collimating a beam to 5–10 µm in combination with a 125 µm × 25 µm focus at the Advanced Photon Source beamline 23ID-B, with data being successfully collected from a sample of 10 µm × 10 µm × 10 µm in size. Recent work has suggested that the use of small samples could limit radiation damage by photoelectron escape (Nave & Hill, 2005 ▶; Cowan & Nave, 2008 ▶).

Initially devoted to small-molecule crystallography, ground-breaking experiments were made on the ESRF beamline ID13 to see if it was feasible to collect data from very small protein crystals. This work allowed one of the early structures of a membrane protein, that of bacteriorhodopsin, to be determined (Pebay-Peroula *et al.*, 1997 ▶; Luecke *et al.*, 1999 ▶) and the advantages of such a method to be evaluated (Cusack *et al.*, 1998 ▶; Cramer & Müller, 1997 ▶). Although it was possible to use the ID13 environment successfully for macromolecular crystallography, the broad range of experiments undertaken on the beamline means that the sample environment was not designed specifically for MX. However, the demand for microfocus MX was such that the ESRF took the decision in 2001 to build a dedicated resource for microfocus macromolecular crystallography.

The design brief of this new beamline was to provide an automated microfocus beamline to enable for the first time the routine and robust use of microfocus MX through the combination of the experience of the ESRF microfocus beamline ID13 with the highly automated and high-throughput environment of the ESRF MX beamlines (Arzt *et al.*, 2005 ▶; Beteva *et al.*, 2006 ▶; McCarthy *et al.*, 2009 ▶). The ID23 facility is made up of two beamlines, each operating as independently from the other as reasonably as possible. The complex has been constructed in two phases. The first-phase beamline, ID23-1 (Nurizzo *et al.*, 2006 ▶), has been operational since early 2004. The second-phase beamline, ID23-2, with a microfocus beam, has been open to the user community since November 2005. This paper describes beamline ID23-2 which is the first dedicated and highly automated high-throughput MX microfocus beamline.

## 2. The ID23 X-ray source

**Figure 1:**
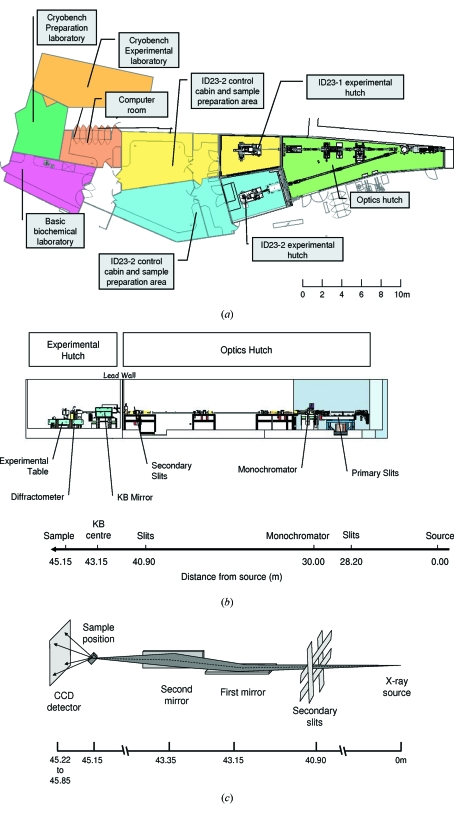
(*a*) Plan view of the ID23 beamline showing the optical and experimental hutches, control cabin and ancillary rooms. (*b*) Schematic showing the layout of the optical elements and experimental hutch of ID23-2 with distances relative to the centre of the straight section which is the smallest beam source point. (*c*) Schematic representation of the beamline focusing. An X-ray camera can be put in front of the detector for beam focusing.

The ID23 X-ray source has been described previously (Nurizzo *et al.*, 2006 ▶) and is a canted undulator system made up of two undulators with an angular separation of 1.5 mrad. The choice of a 1.5 mrad separation was governed by the need to separate the two beams sufficiently to pass through two distinct diamond front-end windows on one hand and the necessity to minimize the design effort involved in modifying the front-end on the other. The canted undulator set-up was chosen to enable the installation of two beamlines on a single straight section, whilst retaining a maximal independence of beamline operation. However, the small separation between the two X-ray beams, some 45 mm at 30 m from the source, creates engineering design challenges. Partly to overcome the space constraints, beamline ID23-2 was constructed as a fixed energy line with a side-deflecting monochromator which allows the two X-ray beams to be separated after the ID23-2 monochromator (Figs. 1 ▶ and 2 ▶) leading to minimal interaction between the two beamlines.

The undulator installed on the ID23 low-β straight section serving beamline ID23-2 is the upstream undulator of the canted system and is of period 20.2 mm, minimum gap 11 mm and length 1.6 m. The device was chosen to give maximum photon output at the beamline working energy of 14.2 keV whilst minimizing heat load. The undulator design therefore generates the primary harmonic at 14.2 keV at the minimum gap setting of 11 mm and corresponding to a *K*_max_ of 0.348 T.

## 3. ID23 beamline design

In the design of ID23, consideration was given to several arrangements for the optical layout: two tunable beamlines, one fully tunable and one partially tunable beamline, and one fully tunable and one fixed-energy beamline. Owing to the relatively low angular separation available from the canted undulators at the time of ID23 construction, the option to build two fully tunable beamlines was not considered tenable in terms of either the floor space available or the budgetary (given non-standard optical components and additional optics to separate the beams, *e.g.* a two-mirror deflection system as used at GM/CA-CAT; Fischetti *et al.*, 2007 ▶) and time constraints involved. The second option of a fully tunable beamline and a second partially tunable line was also ultimately rejected owing to the difficulty in moving large and heavy equipment on a long lever arm to track the beam from a single-bounce monochromator (which would give the spatial separation of the two beams). It was also felt that the limited energy range achieved with this approach did not justify the operational difficulties that would be likely to be encountered with such a system. Finally, the simplest of the options considered was chosen with a fully tunable beamline (ID23-1) and a fixed-energy beamline (ID23-2) using a laterally deflecting monochromator to realise X-ray beam separation. This configuration was possible because the MX beamlines at the ESRF are operated as a unit with rapid flexible access possible to any one of the six MX facilities. Thus the ID23 complex (Fig. 1*a* ▶) strengthens the suite of MX beamlines (two fully tunable and two fixed energy) by the addition of one tunable beamline and one beamline dedicated to microfocus applications with a design goal of achieving a 5–10 µm-diameter focused beam. The decision to fix the energy at 14.2 keV allows anomalous diffraction data to be collected from all the most commonly used elements in MX permitting the beamline to serve a wide range of experiments including *de novo* structure determination.

## 4. Optical design of ID23-2

The principle goal of the ID23-2 beamline optics is to produce a high beam stability, both spatially and temporally with high reliability. To achieve this, the optical design was kept as simple as possible and the number of motors was minimized to reduce the possibility of instabilities introduced by increased degrees of freedom. The optical elements are high-power primary slits, a single-crystal monochromator, a set of secondary slits and a Kirkpatrick–Baez (KB) mirror pair as the focusing element (Figs. 1*b* and 1*c* ▶). A summary of the ID23-2 beamline parameters is given in Table 1 ▶.

### 4.1. Monochromator

The monochromator is composed of a liquid-nitrogen-cooled Si(111) instrument using one laterally diffracting crystal. The monochromator is located at 30 m from the centre of the ID23 straight section and at 15 m from the sample, and the crystal is operated at a nominal Bragg angle of 8.00° with respect to the X-ray beam. The energy selection from the undulator pink beam is made by a combination of monochromator silicon crystal absolute angle, beam divergence, monochromator acceptances and ultimately the secondary slit position just upstream of the focusing elements. The secondary slits define the beam that is incident on the KB mirror pair, reducing the fan of radiation coming from the single-bounce crystal and consequently selecting the final energy range. The single beam deviation of the monochromator and the long lever arm to the sample position means that the stability of the monochromator is critical to successful operation of ID23-2. The number of motorized adjustments in the instrument has therefore been kept to an absolute minimum; as a consequence the Bragg angle of the crystal is aligned at ambient temperature and then bolted to a flexure hinge with a range of ±5 mrad with 50 nrad angular resolution using a stepper motor. This is the only monochromator motor which is used routinely during beamline operation. One advantage of a fixed-energy beamline is that the undulator can be designed specifically to provide the fundamental harmonic at the beamline design energy, thereby reducing the power absorbed in the monochromator crystal. In normal operating conditions a total of 35 W are absorbed on the crystal. This limited absorbed power allowed a cooling system to be designed to minimize vibrations in the crystal rather than to be optimized for thermal efficiency. All undulated tubes in the liquid-nitrogen system were avoided as far as possible and inside the monochromator vacuum vessel only rigid copper tubes are used; the small angular adjustment of crystal Bragg angle is achieved by deforming these annealed tubes.

**Figure 2:**
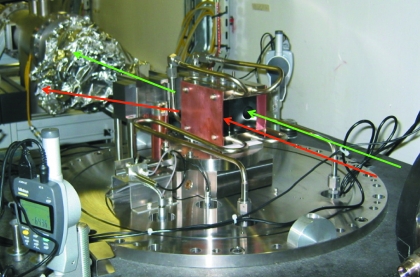
The liquid-nitrogen-cooled silicon monochromator crystal being mounted on the beamline. The vacuum chamber is open. The green arrows indicate the beam path for ID23-2 and the red arrows for ID23-1 through the hole towards the back of the silicon crystal.

The silicon crystal block must allow the ID23-1 white X-ray beam to pass through a hole in the ID23-2 monochromator crystal (Fig. 2 ▶). The liquid-nitrogen tubes feeding the monochromator are flexible pipes, but have been manufactured with an internal braid to maintain laminar flow. Vibration measurements and analysis of beam intensity variations have shown that the liquid-nitrogen flow does not cause any vibrations on the monochromator.

Vibration of any equipment can be particularly damaging to the performance of a microfocus beamline. In order to minimize the propagation of vibrations to the monochromator, the support for the monochromator was constructed using well proven technology developed at the ESRF. A steel reinforced concrete base is poured *in situ*, effectively raising the floor by 920 mm. The monochromator vacuum vessel is rigidly bolted to a granite block which is in turn positioned on the concrete on top of four double-taper *z*-adjustment blocks (Airloc KSKC2150 typically used for supporting machine tools). A high rigidity is maintained by means of preconstraining springs. Two of the *z*-adjustment blocks are motorized to allow adjustment of the tilt of the silicon crystal. This allows the adjustment of the height of the beam at the downstream focusing elements. Since the beamline was commissioned, this freedom has been very rarely used.

### 4.2. Focusing

**Figure 3:**
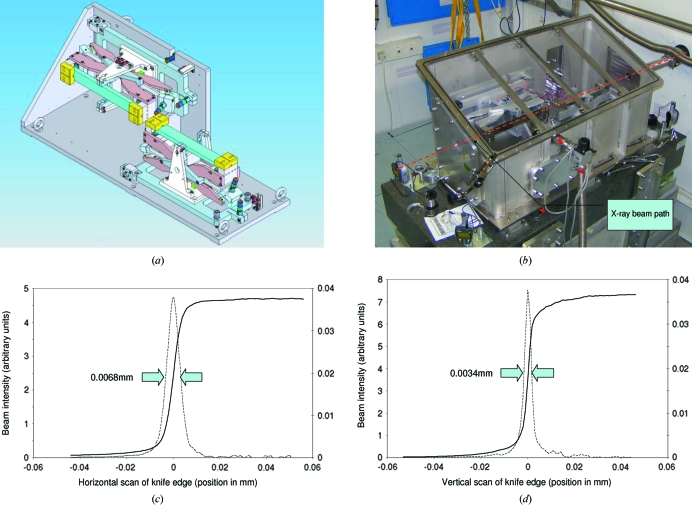
(*a*) Schematic of the ID23-2 KB system. The X-ray beam enters from the right-hand side of the system (vertical focusing first). (*b*) Photograph of the KB system as installed in the experimental hutch of ID23-2. Beam goes from right to left. The KB box has a transparent lid fitted for the photograph. Under normal operation a steel lid is in place, with an over-pressure of helium in the box and the perspex temperature fluctuation damping box surrounding the steel vessel. (*c* and *d*) Knife-edge scans of the horizontal and vertical beam, respectively. The solid line shows the raw data and the dashed line shows the derivative giving the FWHM beam sizes.

The KB mirror architecture (Kirkpatrick & Baez, 1948 ▶) was chosen as the focusing device, consisting of two orthogonal reflecting surfaces with elliptical shapes. The adaptive system, shown in Figs. 3(*a*) and 3(*b*) ▶, is similar to systems previously employed at the ESRF (Eng *et al.*, 1998 ▶; Hignette *et al.*, 2001 ▶, 2003 ▶). Composed of two 300 mm-long platinum-coated silicon mirrors, the horizontal and vertical mirrors focus at a distance of 1.8 m and 2.2 m, respectively, from the centre of the mirror concerned. An X-ray-sensitive camera positioned at the sample position is used to align the system. The central point of the KB pair is situated at 2.00 m from the sample position and at 43.15 m from the source point. Four actuators (designed and manufactured by ESRF) bend the flat polished mirrors into the stigmatic elliptical figures required for imaging the synchrotron source (Zhang *et al.*, 1998 ▶).

The focused X-ray beam that results has been measured to be regularly less than 7.5 µm (horizontal FWHM) by 5.0 µm (vertical FWHM) depending upon the filling mode of the storage ring; the smallest achieved beam size was measured to be 6.8 µm (horizontal FWHM) by 3.4 µm (vertical FWHM) (Figs. 3*c* and 3*d* ▶) when the storage ring is operated in 7/8 multi-bunch mode (the source emittance is increased slightly when the ring is operated in timing modes to improve the lifetime of the stored beam). The beam size was measured using the knife-edge technique by scanning a 200 µm-diameter tungsten wire located at the sample position on the diffractometer ϕ axis vertically or horizontally through the X-ray beam. The beam size and shape are stable over weeks of operation with optimization carried out only occasionally, for example after a ring shutdown as part of the usual beamline checks during the restart. The procedure typically takes about 1–2 h and employs the tools for wavefront analysis developed within the ESRF SciSoft Group (Hignette *et al.*, 1997 ▶; Pieritz & Svensson, 2007 ▶).

As with the monochromator, the stability of the focusing element is critical to the reliable use of microfocus beamlines. The assembly shown in Fig. 3(*a*) ▶ has the possibility of adjusting the height of the vertical focusing mirror and the horizontal position of the horizontally focusing mirror. In addition, and as previously stated, it is possible to steer the beam in both the horizontal and vertical directions with the monochromator. As a consequence it is possible to fix the assembly, shown in Fig. 3(*b*) ▶, in space using conventional alignment techniques foregoing the need for motorized adjustment. A support similar to that of the monochromator has been constructed where the mirror assembly is bolted rigidly to the granite interface and mechanically isolated from the helium enclosure by means of edge-welded bellows.

## 5. Experimental equipment and sample environment

**Figure 4:**
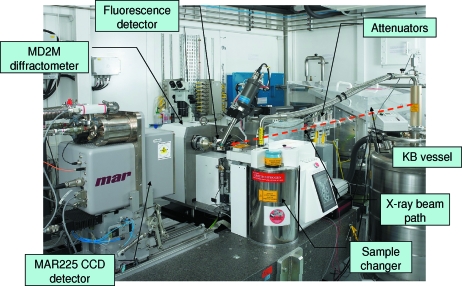
Photograph of the ID23-2 experimental hutch showing the main pieces of equipment. The equipment is the standard ESRF MX experimental set-up consisting of a sample changer, a high-precision diffractometer, a set of attenuators and a large CCD-based detector.

The ESRF MX suite of beamlines makes use of a highly automated and common user hardware and software environment, built on the knowledge gained over the last years of operation (Arzt *et al.*, 2005 ▶; Beteva *et al.*, 2006 ▶). Although ID23-2 is a specialized microfocus beamline, this philosophy has been continued in order to maintain the familiarity and user-friendly environment of the MX beamlines. The experimental table is a block of granite of 2200 mm × 960 mm × 280 mm mounted on three vertical legs plus two horizontal translation stages allowing a total of five degrees of freedom. This table supports the slit box, the diffractometer and the CCD-based detector (currently a MAR 225), all of which are fixed to the table (Fig. 4 ▶). The existing standard ESRF MX beamline set-up of a single-axis diffractometer (a MAATEL/ACCEL mini-diffractometer MD2M, equipped with an additional *YZ* translation stage), with a measured sphere of confusion of 0.8 µm peak-to-peak, and an SC3 sample changer, as developed by the ESRF and the EMBL and commercially available from MAATEL (Cipriani, 2006 ▶), has been replicated in the ID23-2 experimental hutch together with all the ancillary equipment. The experimental hutch also houses the KB mirror pair in a steel container under helium gas maintained at a slight over-pressure.

Details of the experimental hutch diffractometer and general user environment can be found in the paper describing beamline ID23-1 (Nurizzo *et al.*, 2006 ▶).

## 6. Beamline optimization following commissioning and first experiments

### 6.1. Beam stability

Beam stability is a critical issue for a microfocus beamline. Typical exposure times range from 100 ms to 3 s and so variations in intensity in the frequency domain of less than 80 Hz can cause problems. Intensity variations can be seen as either pure intensity variations (caused, for example, by drift-off of the rocking curve of the monochromator crystal or by storage ring instabilities) or positional variations which lead to the sample being exposed in different places during the rotation.

As described in §4 and §5, a number of precautions were taken to minimize possible sources of instabilities. This section describes how successful these precautions have been to date.

#### 6.1.1. Frequency domain > 1 Hz

**Figure 5:**
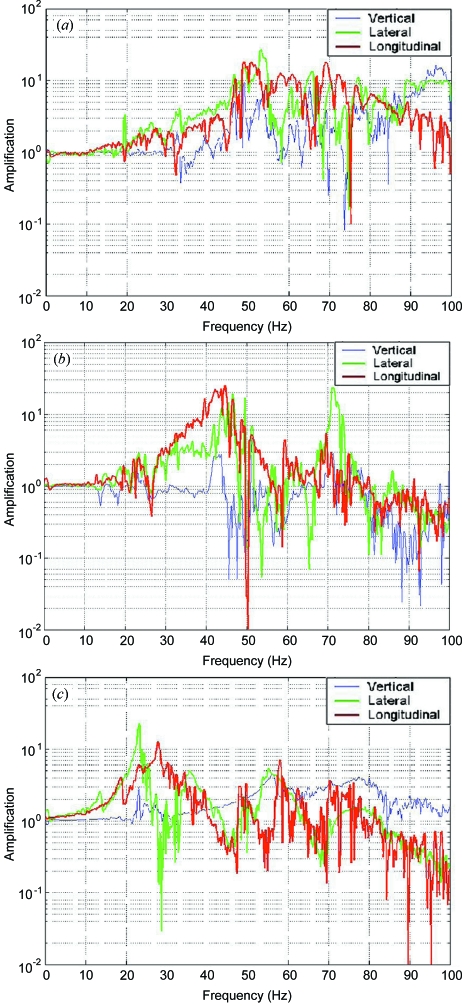
Frequency response of the monochromator support (*a*), the KB system (*b*) and the experimental table (*c*) with respect to the floor. The blue lines show the amplification in the vertical direction, the red lines the amplification in the longitudinal direction along the X-ray beam, and the green lines the amplification in the horizontal plane perpendicular to the X-rays.

The influences of vibrations on beam stability have been carefully checked by regular measurements along the beamline during the construction, commissioning and operation phases. Horizontal and vertical L4-C geophones were located on the selected critical structures: the top of the monochromator vessel, the top of the KB marble, the top of the experimental table marble. Additional sensors on the floor provide references for the vibration levels. Fig. 5 ▶ shows the vibration amplification with respect to the floor on the various elements. The monochromator on its concrete base is very rigid and thus starts to amplify floor motion only from the 50 Hz range. The spectral amplification on the KB system shows that vertically the first vibration mode is located around 70 Hz whereas horizontally the structure is slightly less rigid with modes at 42 Hz and 45 Hz in the beam direction and normal to the beam direction. Amplification factors with respect to floor motion are relatively small and thus the KB structure performs well as far as vibration stability is concerned. The experimental table has a significant amplification starting at 20 Hz and lacks stiffness. This can be attributed to its five degrees of freedom and the relatively large distance between its point of contact on the floor and the sample position. Using a pin diode mounted on the experimental table, it was possible to monitor simultaneously X-ray beam intensity and the vibration levels on the monochromator, the KB marble and the experimental table. Results show little correlation of the intensity with vibrations on the monochromator or KB vessels but more with the experimental table.

A separate system has been used to continuously monitor vibrations and intensities during real beamline operation (Mairs *et al.*, 2006 ▶). It has confirmed the overall performance of the beamline in terms of vibration and has shown the influence of external sources of vibrations such as experimental hutch door closing or people walking around the experimental table or KB support.

**Figure 6:**
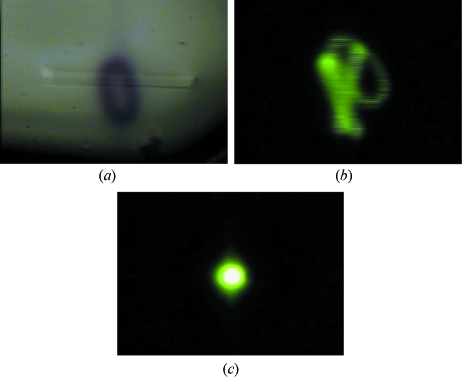
(*a*) The beam mark on the cryoprotectant around the crystal is larger than expected as caused by beam movements in turn resulting from building work on the ESRF site. (*b*) Snapshot of an instant of the moving beam as monitored on the integrated YAG showing the beam trace owing to the perturbations. (*c*) An equivalent beam snapshot using the YAG but under normal (stable) conditions.

Although specific care has been taken to minimize the influence from local vibration sources, unexpected events remote from the beamline can have an influence. For example, beam perturbations owing to building works have been observed once. The works were located inside the ESRF ring approximately 200 m away from ID23-2. The phenomenon was first noticed by chance through an oval beam footprint left in the cryoprotectant around a crystal from which data were being collected (Fig. 6*a* ▶). Owing to the site disturbance, the beam was moving vertically and horizontally, although more vertically. To check the extent of movement, the beam was observed using the integrated scintillator (see below) (Fig. 6*b* ▶), clearly showing significant movement beyond the normal operating status (Fig. 6*c* ▶). Once the building works ceased, the beam returned to its high-stability norm.

#### 6.1.2. Frequency domain < 1 Hz

(i) *Monochromator*. During the commissioning of the beamline a correlation between horizontal beam drifts and ring current was observed which, upon investigation, appeared to be associated with cooling of the side-diffracting monochromator crystal. It was suspected that the liquid-nitrogen-cooled copper blocks were not in optimal thermal contact with the monochromator crystal. Therefore, it was decided to re-design the monochromator crystal and its mechanical support in order to improve the cooling and the crystal support. The original design of the monochromator was foreseen to have two diffracting surfaces, one with an asymmetry of 3° and one symmetrical. The asymmetric option was suppressed in the second version and, as a direct consequence of this, the polishing of the diffracting surface was improved (it could be performed by machine). The updated monochromator crystal support and cooling was installed in October 2005. Since then, no significant beam drifts correlated with the ring current (and the related heat load on the monochromator) have been observed.

**Figure 7:**
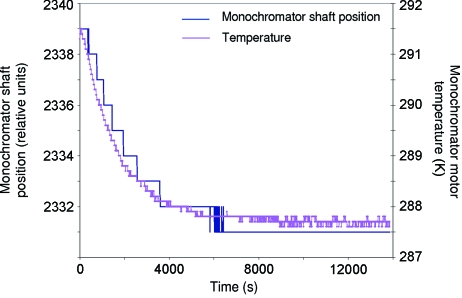
Correlation between monochromator motor temperature and shaft position. Before time 0, the motor underwent several large movements to increase its temperature. The shaft translation was monitored with a linear variable differential transformer. The position became stable after 6000 s.

**Figure 8:**
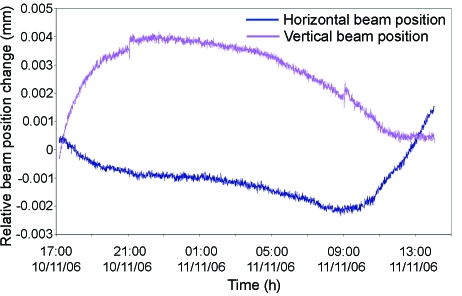
Beam position at the sample position and hutch temperature *versus* time showing the effect of the perspex box to damp the temperature around the KB vessel.

Nonetheless, beam stability was still affected by large horizontal drifts in the range of 50 µm over 1 h. As the drift was occurring in the horizontal plane, it was likely to be also linked to the monochromator. Regular beam-position monitoring and an increase in the available diagnostic tools around the monochromator (an additional thermocouple on the monochromator motor and a linear variable differential transformer connected to the beamline control system) allowed the source of these beam drifts to be identified as being associated with variation of the temperature of the monochromator θ axis motor (Fig. 7 ▶). It was shown that use of the θ motor introduced a temperature increase of the motor and probably the actuator shaft itself. As the motor is housed under vacuum, inside the monochromator vessel, the actuator is effectively insulated leading to small temperature variations with a very long transitional time. Calculations show that the 40 mm actuator of the monochromator θ axis would vary in length by 0.8 µm K^−1^. If this change in body length directly moves the θ axis flexure, a beam movement of some 104 µm could be expected at the sample position. By switching off the motor control card between movements, the motor temperature could be maintained, providing a stable beam. The beam position is checked by a scan of the horizontal secondary slits. If the measured beam position does not correspond to the (known) horizontal mirror entrance, the monochromator angle correction is calculated and applied. As a result, the use of the monochromator motor is minimized and the motor control card is switched off automatically between two movements. Since this methodology has been implemented, the monochromator is re-aligned about once a week. Although the beam drifts correspond to a change in the monochromator diffraction angle, the beam intensity at the sample position also drifts since the secondary slits are not moved.

(ii) *Long-period small-distance beam drifts*. Small beam drifts over a long period of time (*i.e.* in the range of 15 µm in 12 h) were measured during the commissioning period, but with the experimental hutch doors closed throughout the test period. An yttrium aluminium garnet (YAG) screen was used to monitor the beam position over time as the centre of mass calculated from the YAG image. Further tests were conducted where the hutch temperature was deliberately adjusted by 0.5 K steps that showed a correlation with vertical beam movements with a change of 38 µm per degree of increased temperature. To improve the thermal insulation of the focusing elements, installed in the experimental hutch, a perspex box has been fitted around the KB vessel on the granite support table. The temperature inside the perspex box is maintained in the range ±0.1 K. The box damps fluctuations in air temperature and limits the influence of opening/closing cycles of the hutch door. To improve the temperature stability of the experimental hutch as a whole, pipes have been mounted to the exhaust of the cryostream and sample changer liquid-nitrogen dewars to evacuate the cold nitrogen gas to the outside of the hutch. A sample changer is available on the beamline and users are systematically advised to use it whenever possible to keep the experimental hutch closed and so to stabilize the temperature as much possible. The result of these efforts is a reduced beam drift of less than 6 µm over a period of 20 h (Fig. 8 ▶) with the hutch doors closed. However, efforts will continue to monitor the effect of the hutch and component temperature on beam stability.

Whatsoever the source of such beam drifts, a system has been put in place to allow a simple and rapid beam position check to be carried out with minimal impact on the experiment. A pop-up YAG screen at the sample position, the sample being moved 1.5 mm up and downstream to be kept in the cryostream flow, allows the beam to be visualized and the centre of mass to be calculated. Using this information the experimental table is moved according to the correction calculated from the centre of the camera which acts as the overall reference position. This procedure is automated and is installed on all of the ESRF MX beamlines.

### 6.2. Specific concerns regarding the sample support and centring on a microfocus beamline

Matching a very small sample with a very small beam is difficult. Technically, the diffractometer motors (the measured sphere of confusion is better than 0.8 µm) can allow a sample of ∼5 µm × 5 µm × 5 µm to be maintained and centred in the small X-ray beam provided by the ID23-2 optics. However, as sample preparation is an important issue in order to minimize the difficulty of the experiment, it is important to take special care with appropriate sample mounting. Previous experience has shown that the use of adapted sample holders is crucial to reduce the undesirable lens effects caused by a large drop of cryo-buffer around the crystal. It is best to use a sample container that matches the crystal size whenever possible. The use of large traditional nylon loops (20 µm-diameter nylon) is to be discouraged as this can increase these effects particularly in the case of viscous cryoprotectants. The use of the smallest nylon loops (50–100 µm large and 10 µm-diameter nylon) is generally appropriate: the smallest nylon diameter helps to minimize the amount of cryoprotectant and the loop is rigid enough owing to the small size. An alternative is the kapton type of sample support matched to the crystal size; this can reduce the amount of cryoprotectant around the sample and allow a better visualization leading to less difficult centring. The choice of the cryoprotectant is also important, being better to avoid the use of viscous liquids where possible to minimize the formation of a large drop around the sample which will then make a correct centring challenging. Excess cryoprotectant should also be removed around the crystal. In order to help the users in their sample preparation, an inverted microscope (ZEISS Axiovert) combined with a micromanipulator is available in the sample preparation room. The beamline standard Leica binocular microscopes can be equipped with a set of lenses to increase their magnification from 128× up to 640×.

The new generation of diffractometer installed on the ESRF MX beamlines provides the user community with a fast and easy crystal centring tool through the commonly called ‘three-click centring’ procedure. The system is very accurate if the user can ‘click’ always on the same section of the crystal. This can become particularly challenging when the crystal is very small, mounted on a too-large cryoloop and/or embedded in a large amount of cryoprotectant. The actual procedure has proved its efficiency on the other ESRF MX beamlines but has reached its limits on ID23-2 for the smallest of samples. New tools, adapted to routine use of a microfocus beamline for macromolecular crystallography, have been developed. For example a ‘multiple click’ (more than three) procedure is available in addition to the original three-click method. A ‘mesh-scan’ has also been implemented allowing a crystal search in a box defined by the user *via* the graphical user interface using a heavily attenuated X-ray beam as monitor. Owing to the small beam size and the quality of the sample observation, it is possible to mount several small crystals in the same loop. A single sample can then be selected with the computer mouse and be translated directly to the beam coordinates, allowing rapid screening without the need of continual sample centring. Such an approach could also be efficient when screening beamline-mountable crystallization wells (Lunde *et al.*, 2008 ▶) and can be used with larger samples where their crystallinity is not homogeneous over all of the volume and the best section(s) need to be found.

The current standard sample support of pin and loop has reached its limits for smaller micrometre-sized crystals, and the use of so-called ‘free-mounting’ systems (Kitago *et al.*, 2005 ▶), removing cryoprotectant, in their current form is un­likely to help in the case of micro-crystals. The entire sample container, and sample location and centring system will have to be reviewed and re-engineered as crystals and beam continue to reduce their sizes. One technique aimed to improve sample absorption, but that would also improve visibility, is the UV laser processing technique to shape samples by removing surrounding material (including loop and buffer), being developed by Kitano *et al.* (2005 ▶).

## 7. Diffraction data collections

The ID23-2 beam can allow multiple (individually short, incomplete) data sets to be collected from several positions along the longest crystal dimension. Two examples are described below.

### 7.1. Example data collection from a micrometre-sized crystal

The small ID23-2 beam offers a good spatial resolution and the possibility to probe crystals larger than the beam. It has proved useful to screen crystals in order to choose the best one prior to collecting data, with the small beam only locally damaging the crystal whilst leaving a sufficiently undamaged volume for further data collection(s).

The case of the data collection from IREM-1 (an inhibitory receptor involved in the functional regulation of myeloid cells) micro-crystals (Dimasi *et al.*, 2007 ▶) can be given as an example. One of the needle-shaped IREM-1 crystals, of approximate crystal dimensions 300 µm × 10 µm × 10 µm, was mounted in a nylon loop. The very first diffraction pattern (without beam attenuation) showed reflections to a resolution of 2.2 Å. Thereafter a rapid decay of the intensity of the reflections in the higher-resolution shells was evident even after a few frames, which was interpreted as an effect of radiation damage. Increased radiation damage compared with standard synchrotron beamlines is expected at ID23-2, given the high intensity of the beam and its very limited area, although, as mentioned earlier, recent studies suggest that very small samples may actually experience decreased radiation damage with higher X-ray energies (Cowan & Nave, 2008 ▶). Owing to the IREM-1 crystal length, it was possible to collect four independent data sets at different positions along the sample. The distance between the different positions was sufficient to start a new data collection from a crystal volume not significantly affected by beam damage. The final merged data set was complete to 2.6 Å resolution with good statistics and resulted in successful phase determination using molecular replacement. Data collection statistics for the four individual data sets and the merged data are shown in Table 2 ▶.

### 7.2. Example ‘helical’ data collection

Given the high flux density on beamline ID23-2, users are advised to use a total exposure time of not more than 30 s at full beam on one sample position. This recommended limit is configured in the *DNA* (Leslie *et al.*, 2002 ▶) and *BEST* (Popov & Bourenkov, 2003 ▶; Bourenkov & Popov, 2006 ▶) packages which provide automated data collection strategies. In order to extend the possibility of collecting data from needle-like and/or radiation-sensitive crystals, we have developed the option to collect a data set on ID23-2 in a ‘helical’ fashion along a crystal.

The user defines two points on the crystal as the start and end points. One single data set is collected during which the crystal is automatically translated between two points so as to move fresh sample gradually into the beam. The decision to translate the crystal is automatically taken during the data collection as a function of the distance between the two user-chosen positions and the overall angular range. The number of images collected from a single point is then constant for the data set. This has the potential to allow a longer exposure per image without significant radiation damage occurring and thereby increasing signal-to-noise ratios.

**Figure 9:**
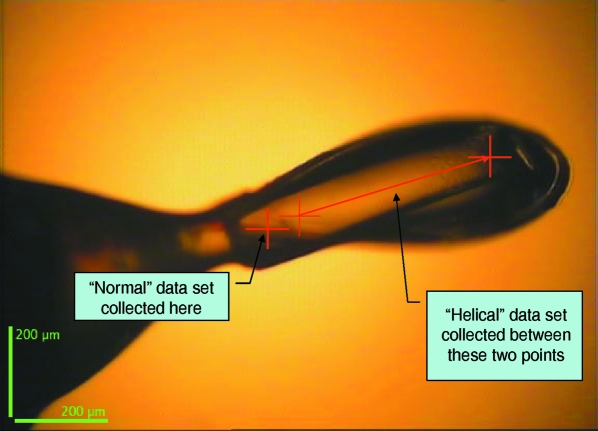
Picture of the crystal used in the helical measurements taken as the X-rays see the sample. The beam size was 7 µm in diameter; the crystal used was approximately 600 µm long by 150 µm wide and 100 µm deep. The normal data set was taken solely around the point on the left of the sample, whilst the helical data set was collected whilst the sample was translated between the two points on the right.

The helical data collection was tested using a crystal of the feruloyl esterase module of xylanase 10B from *Clostridium thermocellum* (Prates *et al.*, 2001 ▶) with selenomethionine residues replacing the natural methionines. Two data sets were collected: one of 270° with 1 s per degree of exposure in a normal mode of collection by rotating the crystal around a fixed point, and a second data set, also of 270°, but where the sample was moved gradually between two points (around 400 µm apart) during the data collection (Fig. 9 ▶) and with a greater exposure of 1.5 s per degree. Data were integrated using *MOSFLM* (Leslie, 1992 ▶) and scaled and converted to structure factors using *SCALA* and *TRUNCATE* (Collaborative Computational Project, Number 4, 1994 ▶). Determination of heavy-atom locations (16 selenium atoms and 10 cadmium ions per asymmetric unit) and phasing were made using the *SHELX* programs (Sheldrick, 2008 ▶) as implemented in *HKL2MAP* (Pape & Schneider, 2004 ▶). Statistics for the two data collections and phasing are shown in Table 3 ▶.

**Figure 10:**
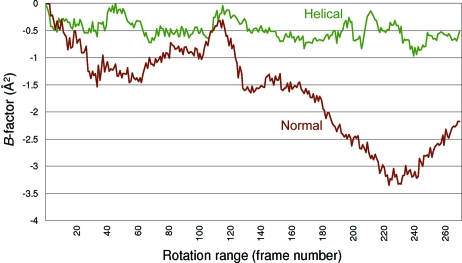
Graph of inter-frame *B*-factors *versus* crystal rotation range (frame) for the normal (rust-coloured line) and helical (green line) data collection methods as extracted from the *SCALA* output file. The normal method shows a steady decrease in the *B*-factors, suggestive of radiation damage to the sample, whilst the *B*-factors for the helical data collection show a far flatter profile.

The results show a clear advantage in using the helical method. Data collected in the traditional fashion have a clear trend in that the inter-frame *B*-factors become steadily more negative, suggesting radiation damage occurring (Fig. 10 ▶). On the other hand, the helical data set has a far flatter profile to the *B*-factors across the rotation range, despite receiving a 50% higher overall dose (with the increased exposure time). As would be expected, the improved signal to noise filters through to all of the data indicators, including the phasing statistics. The helical method of data collection will be described in more detail as the subject of a forthcoming paper, analysing its effects on the resulting data.

## 8. Conclusions and perspectives

Beamline ID23-2 is the first fully dedicated microfocus beamline for macromolecular crystallography. It has been operational since November 2005. The two first structures (Protein Data Bank codes 2CO1 and 2CO2) from data collected on ID23-2 were deposited in June 2006 (Remaut *et al.*, 2006 ▶) and, in the short operation life of ID23-2, 102 structures have been deposited in the Protein Data Bank. Amongst them, highlights include progress made in the knowledge of photosystem I (Amunts *et al.*, 2007 ▶), the calcium transport by the calcium pump (Olesen *et al.*, 2007 ▶) and the human adrenergic G-protein-coupled receptor (Rasmussen *et al.*, 2007 ▶; Warne *et al.*, 2008 ▶).

The successful operation of the beamline using essentially the standard ESRF MX environment and software has shown that microfocus beamlines can be made simple to use. The use of microfocus beams has already been highlighted with work on ESRF beamline ID13. Even if a comfortable use of a microfocus beam has been already achieved on ID23-2, we are nonetheless looking to future improvements. We are working on more sophisticated assisted crystal centring options and automation of data collection for small crystals, particularly for the case when several are randomly oriented in the same sample container. Further microfocus beamlines are planned or under construction at many synchrotrons around the world, including SPring-8 and Photon Factory (Japan) and Petra III (Germany). The ESRF is also considering construction of a microfocus MAD beamline.

The routine beam size on ID23-2 is less than 8 µm (FWHM) but the future demands even smaller beams. One path to help these developments is to use beamline ID23-2 as a test environment to evaluate the efforts and results of using a beam of less than 1 µm in diameter in the context of macromolecular crystallography. The challenge will be to locate, centre and pin the beam onto the sample. Work is currently underway in this direction.

## Figures and Tables

**Table 1:** Summary of beamline ID23-2 parameters

X-ray source	1.6 m-long 20.2 mm-period undulator with 11 mm minimum gap as part of a canted undulator pair serving the ID23-1 and ID23-2 beamlines.
	Source size: 139 µm × 20 µm (FWHM)
	Source divergence: 212 µrad × 7 µrad (FWHM)
Monochromator	Si(111) single crystal operating at 14.2 keV
Focusing element	KB Si substrate mirror pair with Pt coating
	Optically active useful area: 300 mm × 40 mm
	Thickness: 15 mm
	Incident angle: 3.9 mrad
	Micro-roughness: 1.2 Å (VF mirror), 1.0 Å (HF mirror)
	Slope error: 0.56 µrad RMS (VF mirror), 0.38 µrad RMS (HF mirror)
Horizontal focusing ratio (distances)	24:1 (43.35 m source to mirror, 1.80 m mirror centre to sample position)
Vertical focusing ratio (distances)	19.5:1 (42.95 m source to mirror, 2.20 m mirror centre to sample position)
Beam convergence (mrad)	0.55 (H) × 0.36 (V)
Typical secondary slit settings	1.0 mm (H) × 0.8 mm (V), which corresponds to the acceptances of the KB mirrors
Measured flux using calibrated photodiode at the sample with 200 mA stored current (photons s^−1^)	4 × 10^11^
Theoretical focus size (FWHM) at the sample position (V × H) (µm)	1 × 5.8
Measured focus size (FWHM) at the sample position (V × H) (µm)	Normal operating conditions less than 7.5 × 7.5, smallest 6.8 × 3.4 (machine mode dependent)
Lowest achievable resolution	70 Å
Highest achievable resolution (circle inscribed in the square detector)	0.86 Å

**Table 2:** X-ray data-collection statistics for IREM-1 IgV-like domain Values in parentheses are for the highest-resolution shell.

Wavelength (Å)	0.873				
Temperature (K)	100				
Space group	*P*3_1_21				
Unit-cell parameters (Å)	*a* = *b* = 54.23, *c* = 72.01				

	Merged	Data set 1	Data set 2	Data set 3	Data set 4
No. of frames	90	20	33	30	7
Resolution (Å)	19.67–2.60	19.67–2.60	19.67–2.60	19.67–2.61	19.67–2.61
No. of observations	19419	4506	7453	6570	1479
Unique reflections	3936	2686	2783	2984	1265
Data completeness (%)	97.7 (95.7)†	65.1 (65.3)‡	68.7 (66.9)‡	76.9 (80.6)§	31.3 (29.4)§
〈*I*/σ(*I*)〉	9.67 (3.2)†	6.02 (2.9)‡	7.04 (2.9)‡	7.34 (3.1)§	3.96 (2.2)§
*R*_merge_¶ (%)	14.2 (38.8)†	12.5 (25.4)‡	19.3 (47.6)‡	16.1 (42.1)§	19.1 (38.6)§
Molecules per asymmetric unit	1				
Crystal solvent content (%)	37.27				

† Higher-resolution shell 2.60–2.70 Å.

‡ Higher-resolution shell 2.60–2.76 Å.

§ Higher-resolution shell 2.61–2.76 Å.

¶ *R*_merge_ = Σ_*h*_Σ_*i*_|*I*_*hi*_ − 〈*I*_*h*_〉|/Σ_*h*_Σ_*i*_ *I*_*hi*_, where *I*_*hi*_ is the intensity of an individual reflection.

**Table 3:** Data collection and phasing statistics for the crystal of feruloyl esterase module of xylanase 10B from *Clostridium thermocellum* comparing normal and helical data collection methods Values in parentheses are for the highest-resolution shell.

	Data set	
	Normal	Helical
Wavelength (Å)	0.873	
No. images/oscillation angle	270/1.0°	
Temperature	100 K	
Exposure time per image (s)	1.0	1.5
Space group	*P*2_1_2_1_2_1_	
Unit-cell parameters (Å)	*a* = 65.7, *b* = 108.8, *c* = 113.6	
Resolution range (Å)	56.89–1.70 (1.74–1.70)	56.89–1.70 (1.74–1.70)
No. unique reflections	89547	89503
Completeness (%)	99.2 (91.8)	99.1 (90.9)
*R*_merge_ (%)†	7.8 (34.0)	4.9 (14.2)
Multiplicity	10.5 (7.3)	10.7 (7.5)
(*I*)/〈σ(*I*)〉	25.2 (5.7)	32.2 (11.5)
Wilson *B*-factor (Å^2^)	11.9	12.3
*SHELXD* best CC all/weak (%)	48.27/28.33	56.59/35.23
*SHELXD* best Patterson figure-of-merit	6.58	8.16
*SHELXE* pseudo-free CC after 20 cycles of density modification (%)	78.9	80.9
*SHELXE* average peak height (σ) corresponding to the 26 heavy atoms (16 Se + 10 Cd)	32.3	38.1

† *R*_merge_ = Σ_*h*_Σ_*i*_|*I*(*h,i*) − 〈*I*(*h*)〉|/Σ_*h*_Σ_*i*_ *I*(*h,i*), where *I*(*h,i*) is the intensity of the *i*th measurement of reflection *h* and 〈*I*(*h*)〉 is the mean value of *I*(*h,i*) for all *i* measurements.
